# Design and construction of cuttlefish ink derived melanin complexed microneedles for microenvironment regulation to improve vitiligo treatment

**DOI:** 10.1007/s42995-025-00318-5

**Published:** 2025-08-06

**Authors:** Weimiao Li, Yan Shi, Ruimin Zhan, Lu Liu, Jiarui Wang, Minhyeock Lee, Bingqiang Zhang, Shaoshuai Liang, Zhiguo Wang, Ming Kong

**Affiliations:** 1 College of Marine Life Science, Ocean University of China, Qingdao, 266003 China; 2 Department of Burn and Plastic Surgery, The Affiliated Hospital of Qingdao University, Qingdao University, Qingdao, 266003 China; 3 Department of Biotechnology, College of Life Sciences and Biotechnology, Korea University, Seoul, 02841 Korea; 4 Qingdao Restore Biotechnology Co., Ltd., Qingdao, 266111 China

**Keywords:** Vitiligo, Microneedle, HMGB-1, Natural melanin, Exosome

## Abstract

Vitiligo is an autoimmune disorder marked by melanocyte destruction and epidermal depigmentation, primarily driven by inflammatory and oxidative stress within the affected skin lesions. Consequently, there is an urgent need for therapeutic strategies focused on protecting melanocytes and replenishing melanin for effective vitiligo management. In this study, a novel microneedle-based therapeutic platform (C/D/E@MN) was fabricated that was composed of cuttlefish ink nanoparticles (CINPs) for melanin supplementation, dipotassium glycyrrhizinate (DPG) for inflammation regulation, and skin-derived exosomes (EXO) to promote melanocyte proliferation. In addition, microneedles with varying dissolution profiles (swellable, slow-dissolving, and fast-dissolving) were designed and evaluated their performance to optimize therapeutic efficacy. In vitro results demonstrated that fast-dissolving microneedles (FDMN) significantly reduced cellular reactive oxygen species (ROS) and the secretion of vitiligo-related inflammatory cytokines and chemokines, such as IL-8, CXCL-16, and HMGB-1. Upon a vitiligo mice model, C/D/E@FDMN treatment group generated a significant increase in skin melanin content and a 15.5% reduction of whitening degree. The microneedles protected melanocytes and promoted lesion repigmentation through synergistic antioxidant, anti-inflammatory and cyto-proliferative mechanisms, offering a promising strategy for improved vitiligo therapy.

**Supplementary Information:**

The online version contains supplementary material available at https://doi.org/10.1007/s42995-025-00318-5.

## Introduction

Vitiligo, an autoimmune disorder, is characterized by chronic pigment loss and the formation of white skin patches, which can significantly impair individuals' self-esteem and even induce anxiety or depression. The pathogenesis of vitiligo is primarily driven by melanocyte destruction, oxidative stress, and a pro-inflammatory microenvironment (Chen et al. 2021). Therefore, strategies such as modulating the inflammatory microenvironment at the site of vitiligo, promoting melanocyte proliferation (Chen et al. 2021), and replenishing exogenous melanin (Sun et al. 2021) hold great potential to improve the vitiligo treatment. Current therapeutic options for vitiligo generally rely on anti-inflammation or anti-immune responses drugs, such as steroids, calcineurin inhibitors, in combination with ultraviolet to stimulate melanocyte regeneration (Picardo et al. 2015). However, these treatments are costly and efficacy-limited requiring to develop a safe, effective and cost-efficient strategy.

Microneedles (MNs) consist of array of micro-sized needles, ranging from 25 to 1000 µm in height and of different shapes, and substrate (Donnelly et al. 2010). MNs are capable of overcoming the stratum corneum barrier to transdermally and efficiently deliver drugs into systemic circulation (McCrudden et al. 2015), leading to favorable drug pharmacokinetics and patient compliance (Meng et al. 2020). Due to the advantages, MNs have been extensively studied and applied in skin disease, such as psoriasis (Qu et al. 2023), eczema (Shen et al. 2024), psoriasis (Du et al. 2019), hypertrophic (de Decker et al. 2023) scars and so on. To address different drug delivery demands, MNs with different characteristics have been fabricated. Fast-dissolving MNs could release drugs instantaneously upon insertion into the skin, while swellable MNs are able to absorb interstitial fluid and form hydrogels to achieve sustained drug release. Slow-dissolving MNs have also been developed (Ma and Wu 2017). Besides transdermal drug delivery, swellable MNs can be used for detecting potential biomarkers in interstitial fluid, such as glucose (Liu et al. 2021) and lactate (Bollella et al. 2019). For vitiligo treatment, formulations of MNs require to be validated to optimize their therapeutic efficacies.

Cuttlefish ink sacs, a source of natural melanin, are often discarded during processing, resulting in resource wastage and environmental pollution (Trigo et al. 2023). Nevertheless, cuttlefish ink nanoparticles (CINPs) own various biological functions, including photoprotection, antioxidant activity, antitumor properties and anti-inflammatory effects (Deng et al. 2019), making it extensively applicated in the biomedical field. Mammalian melanin, a high-molecular-weight pigment produced through the oxidative polymerization of phenolic compounds or indoles by tyrosinase, is composed of heterogeneous molecules presenting two forms: dark eumelanin and yellow pheomelanin (Xie et al. 2021). Cuttlefish ink melanin is a copolymer of eumelanin composed of two sorts of moieties, 5,6-dihydroxyindole (DHI) and 5,6-dihodroxyindole-2-carboxylic acid (DHICA) derived from tyrosine (Caldas et al. 2020). Based on the chemical homology between cuttlefish ink melanin and mammalian melanin, it is hypothesized that CINPs could improve vitiligo treatment by modulating the inflammatory microenvironment and replenishing exogenous melanin.

High mobility group box-1 protein (HMGB-1) is an extracellular pro-inflammatory or chemotactic molecule. Through binding to its receptors, such as TLR4, the NF-κB pathway is activated to produce inflammatory cytokines, such as TNF, IL-6 and IFN-γ, etc. (Tao et al. 2015). The HMGB-1 content in the blood of vitiligo patients was significantly elevated (Kim et al. 2017), and the HMGB-1 expression in vitiligo lesion was significantly up-regulated compared with the normal skin (Yang et al. 2013). Glycyrrhizin acid can directly bind to HMGB-1 and inhibit its extracellular pro-inflammatory activity (Vitali et al. 2015). Exosome (EXO) is an extracellular secretion released by cells, which contains active molecules that could regulate cell growth, differentiation, apoptosis, etc. (Kalluri and LeBleu 2020). EXO could participate in intercellular communication as well as cell repair and regeneration by activating the Wnt/β-catenin signaling pathway (Tan et al. 2024). EXO derived from the skin tissue is assumed to exert positive effects on proliferation and migration of melanocytes.

**Fig. 1:**
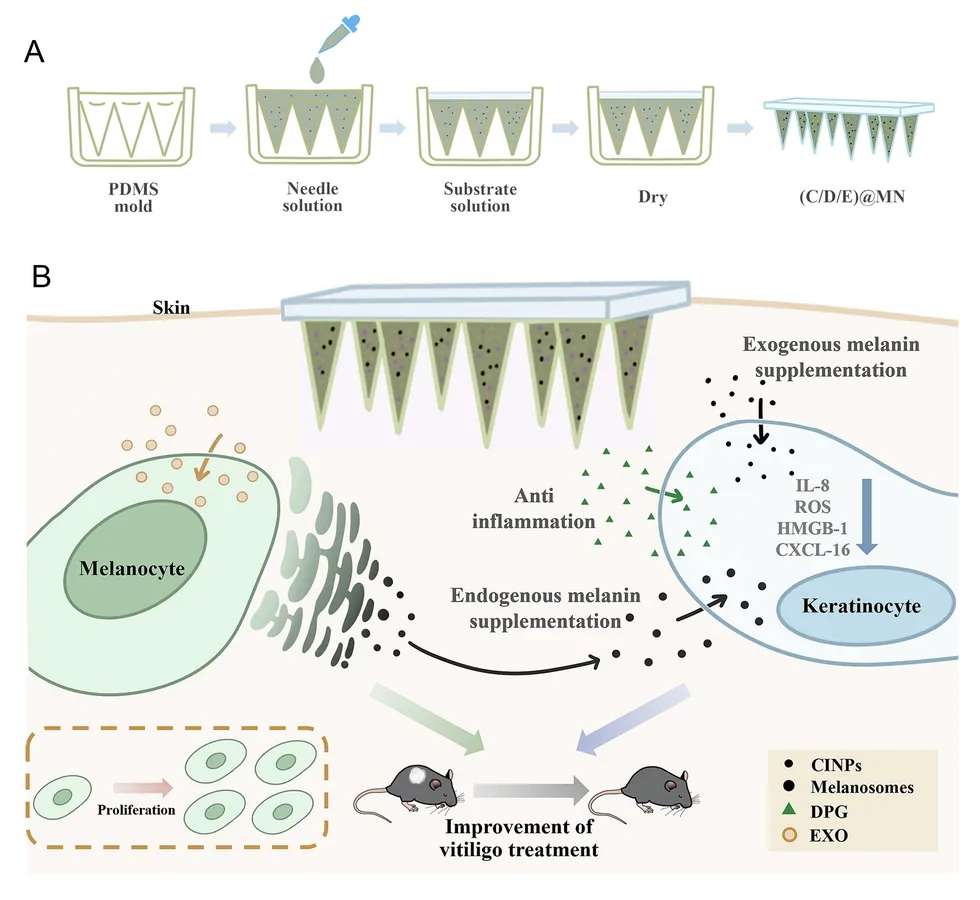
Schematics of microneedle fabrication and mechanism of vitiligo therapy. **A** Flow chart of microneedle fabrication. **B** Transdermal delivery of CINPs (cuttlefish ink particles), DPG (dipotassium glycyrrhizinate) and EXO (exosomes derived from mouse dorsal skin cells) in vitiligo lesion and mechanism for improving therapy

In this study, a novel complexed microneedle, consisted of CINPs, DPG and EXO, was fabricated to modulate the vitiligo microenvironment. Different formulations of microneedle were compared to optimize drug release performance. Therapeutic efficacies were studied using oxidative stress cellular model and vitiligo mice model. The vitiligo treatment was expected to be improved via the synergetic effects of melanin supplementation, anti-inflammation and melanocytes protection (Fig. 1).

## Materials and methods

### Materials and animals

Cuttlefish (~ 500 g) were purchased from Xinjie Marine Products Co. Ltd. (Qingdao, China). *C57BL/6* mice (male, 5 g, 3 weeks) were purchased from Pengyue Experimental Animal Breeding Co. Ltd. (Jinan, China). DPPH free radical scavenging assay kit, total antioxidant capacity assay kit (ABTS method), Reactive Oxygen Species Assay Kit with CM-H2DCFDA were purchased from Beyotime Biotechnology Co. Ltd. (Shanghai, China). PVP (K-60), high alcoholysis PVA (HPVA) (alcoholysis: 97.5–99.0 mol%), low alcoholysis PVA (LPVA) (alcoholysis: 92.0–94.0 mol%) were purchased from Aladdin Biochemical Technology Co. Ltd. (Shanghai, China). Hyaluronic acid was purchased from Bloomage Biochemical Technology Co. Ltd. (Beijing, China). Dipotassium glycyrrhizinate (DPG) was purchased from Macklin Biochemical Bechnology Co. Ltd. (Shanghai, China). Dulbecco’s modified eagle medium (DMEM), minimum essential medium (MEM), Cell Counting Kit-8 (CCK-8), Phosphate buffered (PBS), fetal bovine serum (FBS) and simulated body fluid (SBF) were purchased from Solarbio Biotechnology Co. Ltd. (Beijing, China).

### Extraction and characterization of CINPs

CINPs were extracted from cuttlefish ink by centrifugation. In brief, frozen cuttlefish were thawed and the ink sacs were dissected to obtain the ink. A 20-fold volume of deionized (DI) water was added to the fresh ink, and the mixture was stirred at 4 ℃ overnight to obtain uniform dispersion. The mixture was centrifuged at 4 °C and 20,000 *g* for 15 min, the precipitate was collected and rinse with DI water again. After repeating the process six times, the precipitate was vacuum lyophilized to obtain CINPs. The chemical structure of CINPs was detected by Fourier transform infrared (FTIR) spectrometer (5DX/550 II, Nicolet, USA). The ROS scavenging ability of CINPs was characterized using DPPH free radical scavenging assay kit and total antioxidant capacity assay kit (ABTS method). CINPs were suspended in DI water and diluted to measure their size distribution and zeta potential using Zetasizer (Malvern Panalytical, Malvern, England). The morphology of CINPs was observed by transmission electron microscopy (TEM, Hitachi H-600, Japan Electron Optics Laboratory, Japan).

### Fabrication of MNs

MNs are fabricated using PDMS molds with pyramid needle height of 600 μm and side length of 300 μm. Swellable microneedle (SMN) was prepared of PVA with different ratio (1:2 and 1:4) of HPVA and LPVA at a total concentration of 20%. LPVA and PVP were used to fabricate slow-dissolving microneedle (SDMN) at a total concentration of 20%. Fast-dissolving microneedle (FDMN) was fabricated of 35% PVP. CINPs, DPG and EXO were added into the needle solutions at concentration of 60 μg/mL, 300 μg/mL and 60 μg/mL, respectively. The needle solution was cast into the needle cavities of PDMS molds and centrifuged at 1800 *g* for 5 min. The excess needle solution was removed and 5% hyaluronic acid (HA) solution was supplemented. After thorough drying at room temperature, the MNs were carefully demolded from the PDMS molds and stored for further use.

### Characterization of MNs

MNs were coated by plasma sputtering and then observed using scanning electron microscopy. The mechanical properties of the MNs were evaluated using a universal testing machine (CTM, Hip Keung Instrument Co. Ltd., China). The MNs were immersed in SBF, the morphological variation of needles and their swelling or dissolving performances were continuously observed and measured. For the release properties of CINPs and DPG, the light absorbance at 257 nm (for DPG) and 450 nm (for CINPs) of the drug loaded microneedle immersion were recorded at various time intervals to establish the drug release profile. The concentrations of CINPs and DPG loaded in MNs are 0.1 mg/mL and 0.5 mg/mL, respectively. MNs loaded with CINPs were inserted into a simulated skin (agarose hydrogel, 3% m/v) or mice dorsal skin to assess insertion capacities. The pinholes with CINPs residue or stained with trypan blue after insertion were observed by microscope (Nikon, Ts2R, NIS Elements). Insertion ratio was calculated by dividing the number of blue staining spots by the number of array needles.

### Internalization of CINPs by keratinocytes (HaCaTs)

HaCaTs were taken as model of keratinocytes to evaluate the internalization of CINPs. HaCaTs were incubated with different concentrations of CINPs or FITC-labeled CINPs for 24 or 48 h. After rinse with PBS, the uptake of CINPs by HaCaTs was observed using fluorescence microscope (Nikon, ECLIPSE Ts2, NIS-Elements) for FITC-labeled CINPs or TEM for CINPs in form of cell sections. In the meantime, the cells were collected and lysed with 0.2 mol/L NaOH solution, the absorbance value at 450 nm of CINPs was measured to quantify the internalization efficiency.

### Antioxidative activity assessment

According to the method described in the Supplementary Information, the oxidative stress cell model was established. Oxidative stressed HaCaTs were co-cultured with different concentrations of CINPs, oxidative stressed HaCaTs without CINPs was taken as positive control (P.C.). Intracellular ROS levels within HaCaTs were labeled by DCFH–DA and observed by fluorescence microscope. Semi-quantitative analysis of concerning ROS fluorescence intensity was performed using ImageJ. The contents of IL-8, HMGB-1 and CXCL-16 secreted by HaCaTs were detected by enzyme-linked immunosorbent assay (ELISA) method.

### In vitro migration and proliferation assay of HaCaTs and melanocytes (MCs)

According to the methods described in *Supplementary Information*, dorsal skin cells and melanocytes were extracted and primarily cultured. The exosomes were extracted from primary culture dorsal skin cells and characterized. The HaCaTs and MCs were inoculated in 6-well plates at a density of 1 × 10^6^ cells/mL at 37 °C under 5% CO_2_, respectively. After 24 h, the medium was discarded and replaced with new medium supplemented with 60 μg/mL CINPs, 60 μg/mL CINPs/300 μg/mL DPG or 60 μg/mL CINPs/300 μg/mL DPG/60 μg/mL EXO, respectively, the untreated cells were taken as the negative control (N.C.). Then the cell monolayer was scraped using a 200 μL pipette tip. Images were taken at different timepoints with a microscope, and the migration rate of the cells was calculated with ImageJ. In addition, cell proliferation assay was measured using cell counting Kit-8 (CCK-8) after 48 h culture.

### Animal experiments

The vitiligo mouse model was established according to previous study (Zhu et al. 2013). Briefly, 40% monobenzone ointment was applied to the shaved dorsal skin of mice once a day. After successful establishment, the vitiligo mice were randomly divided into five groups (*n* = 5), namely, vitiligo positive control group (P.C.), tacrolimus treated group, (C/E)@SDMN group, (C/D/E)@SDMN group and (C/D/E)@FDMN group. All animal experiments were conducted in compliance with local guidelines and approved by the Animal Care and Use Committee of the Ocean University of China. The dorsal skin hair of the mice was removed and the exposed dorsal skin was treated with different formulations on days 0, 2, 4, 6, 8, 10 and 12. On days 0 and 14, the treated areas were photographed for repigmentation assessment. On day 14, the weight of the mice was recorded, and the treated dorsal skin of mice was sampled for histological section analysis using Masson–Fontana staining or immune staining with high mobility group box-1 (HMGB-1) protein fluorescence antibody.

### Statistical analysis

Data were expressed as mean ± SD. Statistics were analyzed by Student's *t* test and ANOVA (Prism5 GraphPad). Significant differences between two groups are marked with an asterisk (n.s.: *P* > 0.05, *P < 0.05, **P < 0.01, ***P < 0.001). All experiments had to be repeated independently at least three times.

## Results

### Extraction and characterization of exosomes derived from mouse skin

**Fig. 2:**
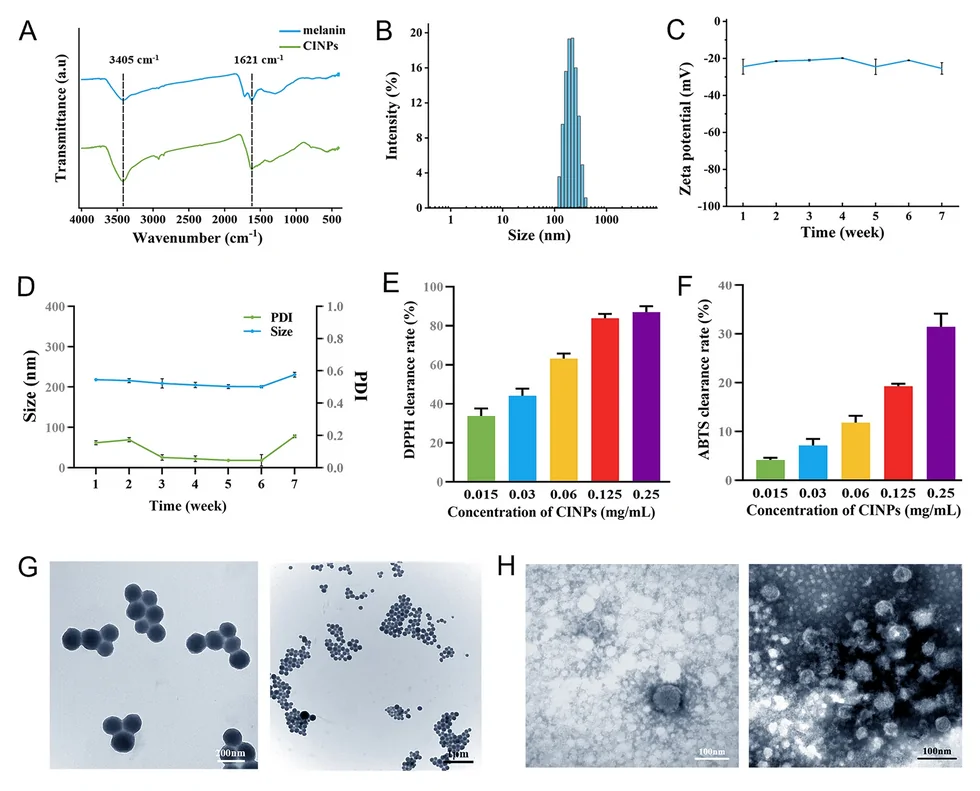
Characterization of CINPs and EXO. **A** Fourier transform infrared spectroscopy of CINPs and standard regent of melanin. **B** Particle size distribution of CINPs. Stability evaluation of CINPs in terms of zeta potential (**C**), particle size and polydispersity index (PDI) (**D**). Antioxidant activities of CINPs characterized by DPPH (**E**) and ABTS (**F**) assay. **G** Representative TEM images of CINPs. **H** Representative TEM image of EXO

To extract EXO, cells were isolated from the dorsal skin of *C57BL/6* mice and cultured according to previous literature (Shi et al. 2022). After 48 h of culture, the cells exhibited a short spindle or triangular morphology and expanded to a larger size with abundant cytoplasm (Supplementary Fig. S1A). By the third passage, the cells appeared long spindle shape and grew in confluent monolayers, the culture medium was collected for EXO extraction. The extracted EXO exhibited spherical morphology with an average diameter of 122 nm (Fig. 2H, Supplementary Fig. S1C) and an average potential of -9.03 mV (Supplementary Fig. S1D).

### Fabrication and characterizations of CINPs

CINPs are easily available natural melanin with photoprotective, antioxidant, anti-inflammatory activities. Given that vitiligo lesions are markedly deficient in melanin, targeted delivery of CINPs is anticipated to ameliorate the inflammatory and oxidative stress microenvironment while supplement exogenous melanin. CINPs were isolated from cuttlefish ink sacs via successive centrifugations and washing with deionized water (Fig. 2G). The yield of CINPs was 5.3%. The chemical structure of CINPs was characterized by Fourier Transform Infrared Spectroscopy (FTIR) and compared with standard melanin reagent. (Fig. 2A). A strong and broad band at 3405 cm^−1^ was attributed to the stretching vibrations –OH and –NH. The peaks at 2917 cm^−1^and 2847 cm^−1^ were correspond to the stretching vibrations of CH_3_ and CH_2_, respectively. A strong absorption peak at 1621 cm^−1^ was assigned for the stretching vibrations of the aromatic C=C and C=O group stretching vibrations. The absorption peak at 1370 cm^−1^ was attributed to the O–H deformation and C–O stretching of the phenolic O–H group. The weak absorption peaks at 800–600 cm^−1^ indicate that some positions of the aromatic ring have been substituted to form a conjugated system with low aromatic hydrogen content. Among these absorption peaks, the peaks at 3405 cm^−1^ and 1621 cm^−1^ were the representative for melanin. CINPs displayed a homogeneous spherical morphology with zeta potential of − 24.4 ± 1.2 mV (Fig. 2C) and hydrodynamic size of 215 ± 52 nm (Fig. 2B). The stability of CINPs was assessed by measuring zeta potential, hydrodynamic diameter and polydispersity index (PDI) over seven weeks, suggesting desirable stability (Fig. 2D).

The antioxidant activities of CINPs were evaluated by DPPH assay kit and ABTS assay kit. The radical scavenging ability of CINPs was concentration-dependent (Fig. 2D, E), with maximum scavenging rates of 81.07% for DPPH and 31.47% for ABTS at 0.25 mg/mL CINPs. To assess the biosafety, the cytocompatibility of CINPs was verified. Live/dead cell staining and relative cell proliferation assays demonstrated that CINPs exhibited good cytocompatibility (Supplementary Fig. S3, S4A). Hemolysis assay was performed using whole blood of New Zealand rabbits at 37 °C for 2 h. Deionized water and saline were used as positive and negative controls, respectively. The hemolysis rates of CINPs were all less than 5%, suggesting no hemolysis occurred under the test concentrations (Supplementary Fig. S4B). The above results demonstrated that CINPs were safe and biocompatible ranging from 3.75 to 60 μg/mL, and 60 μg/mL was taken for the following experiments.

### Internalization of CINPs by HaCaTs

**Fig. 3:**
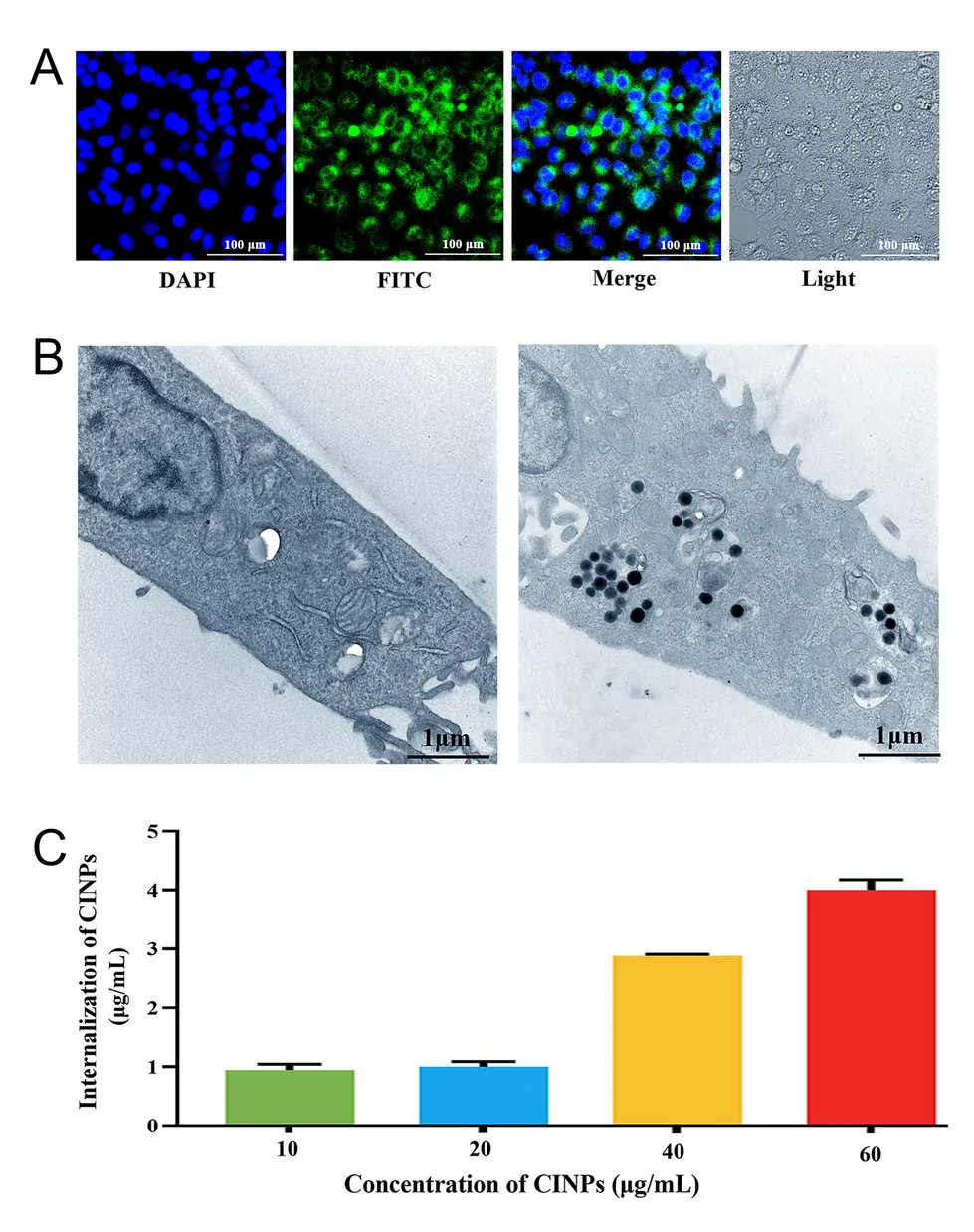
Internalization of CINPs by HaCaTs. **A** Representative laser confocal microscopic images of FITC-labeled CINPs internalized by HaCaTs. **B** Representative TEM images of cell section of HaCaTs co-incubated with or without 60 μg/mL CINPs. **C** Intercellular content of CINPs in HaCaTs after co-incubation with different concentrations of CINPs for 24 h

In living organisms, melanin is produced by epidermal MCs and transferred to the surrounding keratinocyte, thus displaying pigmentation on the skin (Wu et al. 2012). To verify the internalization of CINPs by keratinocytes, HaCaTs were applied as model cell. After co-incubating HaCaTs with 60 μg/mL FITC-labeled CINPs for 24 h, green fluorescence was found around the nucleus, suggesting that CINPs could be internalized by HaCaTs (Fig. 3A). This was also confirmed by cell section observation (Fig. 3B), where CINPs were mainly found in cytosolic vesicles. Moreover, intracellular CINPs contents were detected by lysing HaCaTs after internalizaiton (Fig. 3C), where the absorbance (450 nm) of the cell lysate was positively correlated with the amount of CINPs. These results validate the efficient internalization of CINPs by HaCaTs, underscoring its potential efficacies in repigmenting vitiligo lesions.

### Oxidative stress model of HaCaTs

**Fig. 4:**
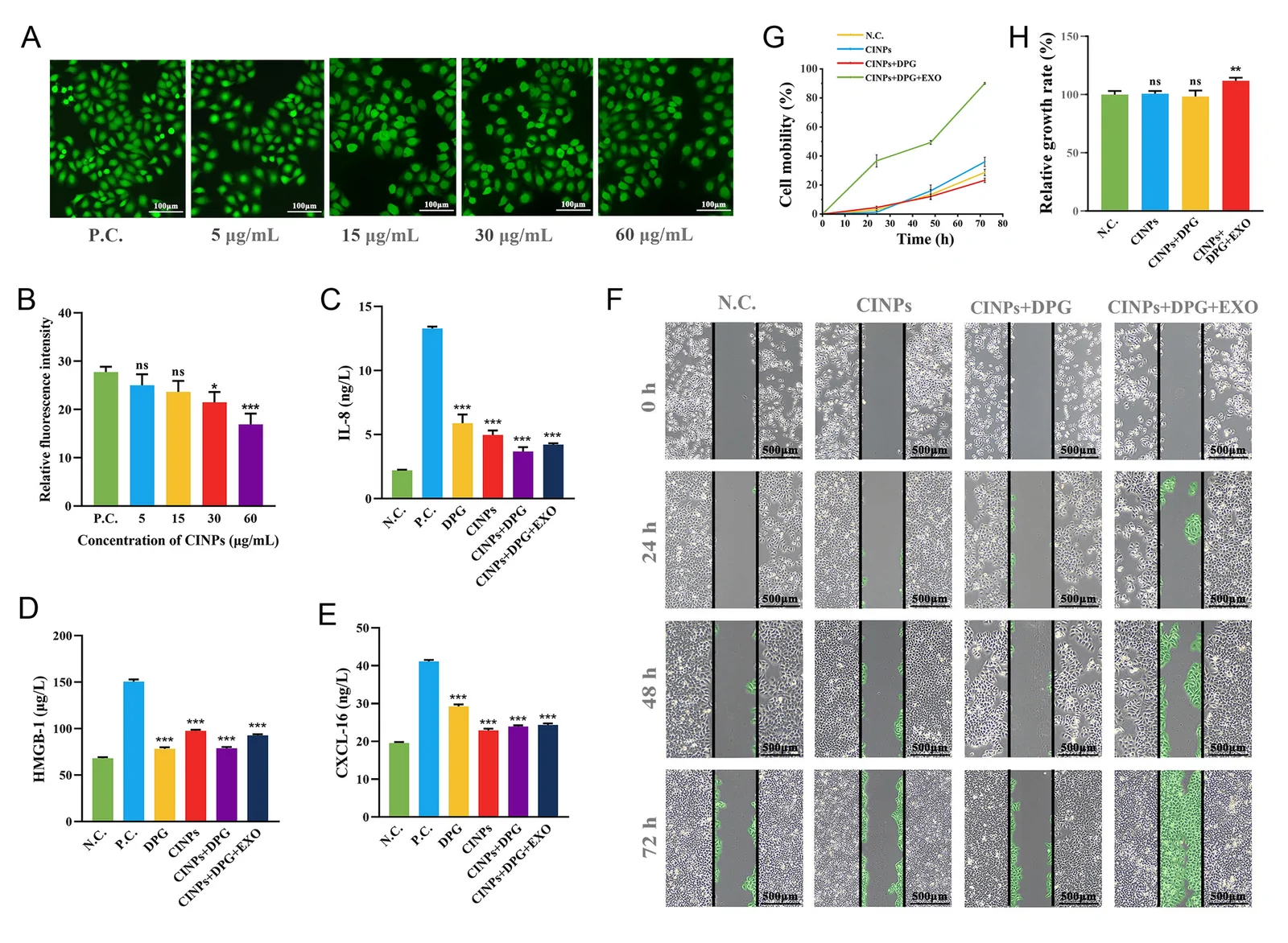
In vitro therapeutic efficacies assessment of active ingredients on oxidative stress HaCaTs model. **A** Representative fluorescence images DCFH–DA labeled intracellular ROS of HaCaTs co-incubated with different concentrations of CINPs. The untreated oxidative stress HaCaTs were taken as positive control (P.C.) **B** Semiquantitative analysis of ROS fluorescence intensities in the microscopic images. The level of IL-8 (**C**), HMGB-1 (**D**) and CXCL-16 (**E**) secreted by the oxidative stress HaCaTs cells after treatment with CINPs (60 μg/mL), CINPs + DPG (60 μg/mL CINPs and 300 μg/mL DPG) or CINPs + DPG + EXO (60 μg/mL CINPs, 300 μg/mL DPG and 60 μg/mL EXO), respectively. The untreated oxidative stress HaCaTs were taken as positive control (P.C.) and the normal HaCaTs were taken as negative control (N.C.). Statistical difference was analyzed between experiment groups and P.C. group. **F** Confluent monolayers of HaCaTs were scratched with a pipette tip and incubated with CINPs (60 μg/mL), CINPs + DPG (60 μg/mL CINPs and 300 μg/mL DPG) or CINPs + DPG + EXO (60 μg/mL CINPs, 300 μg/mL DPG and 60 μg/mL EXO). The untreated oxidative stress HaCaTs were taken as N.C. **G** Quantitative analysis of the decrease in scratching area of HaCaTs. **H** Relative growth rate of HaCaTs incubated with CINPs (60 μg/mL), CINPs + DPG (60 μg/mL CINPs and 300 μg/mL DPG) or CINPs + DPG + EXO (60 μg/mL CINPs, 300 μg/mL DPG and 60 μg/mL EXO) for 24 h. The untreated oxidative stress HaCaTs were taken as N.C. Statistical difference was analyzed between experiment groups and N.C. group. Data are expressed as the mean ± SD (*n* = 3), *p* values are calculated using one-way ANOVA with Bonferroni correction, n.s.: *P* > 0.05, *P < 0.05, **P < 0.01, ***P < 0.001

An oxidative stress model of HaCaTs was established by H_2_O_2_ at a half lethal concentration of 150 μmol/L (Supplementary Fig. S5). Different representative factors including IL-8 (inflammatory factors), CXCL-16 (chemokines) and HMGB-1 (pro-inflammatory factors) secreted by HaCaTs were determined, respectively. The ROS levels in HaCaTs co-cultured with CINPs decreased in a concentration-dependent manner and were significantly lower than those in the control group (Fig. 4A, B), around 10% decrease of DCFH–DA positive cells were observed after treatment with 60 μg/mL CINPs compared with the P.C.. The levels of IL-8, HMGB-1, and CXCL-16 in HaCaTs co-cultured with DPG, CINPs, EXO or their combinations also significantly reduced (Fig. 4C–E). DPG produced the lowest HMGB-1 levels due to its specific inhibitory effect, however, the additional inclusion of CINPs did not further reduce HMGB-1 expression. CINPs significantly downregulated IL-8 expression, and its combination with DPG achieved the optimal effect, demonstrating their synergistic efficacy. Similar trends were observed in the CXCL-16 measurements. In contrast, EXO did not produce a statistically significant improvement. The results confirmed the in vitro antioxidant and anti-inflammatory activities of CINPs and DPG, which were desirable for reversing the vitiligo microenvironment.

### Proliferation and migration of HaCaTs and MCs

Vitiligo is an autoimmune depigment disease results from extensive MCs destruction. Therefore, it is crucial to restore the number and function of the MCs resident in lesions, and recruit healthy MCs around. Dorsal skin cells were extracted from *C57BL/6* mice, enriched and then centrifuged to obtain EXO (Rahmani et al. 2014). To assess the effects of different ingredients, blank control group, CINPs group, CINPs + DPG group, and CINPs + DPG + EXO group were set up for investigation. In vitro migration assays demonstrated that neither CINPs nor DPG significantly affected the migration or proliferation of HaCaTs or MCs, but EXO significantly promoted the cells migration and proliferation. Specifically, the migration rate in the EXO-treated group was 4.3-fold higher for HaCaTs (Fig. 4F, G) and 2.2-fold higher for MCs (Supplementary Fig. S6A, B) compared to the control group. Furthermore, EXO increased the proliferation rate of HaCaTs by 12.1% (Fig. 4H) and that of MCs by 24.3% (Supplementary Fig. S6C). These findings suggested that skin-derived exosomes effectively promoted the migration and proliferation of HaCaTs and MCs, thereby increasing MCs numbers and endogenous melanin content in vitiligo lesions.

### Fabrication and characterization of microneedles

**Fig. 5:**
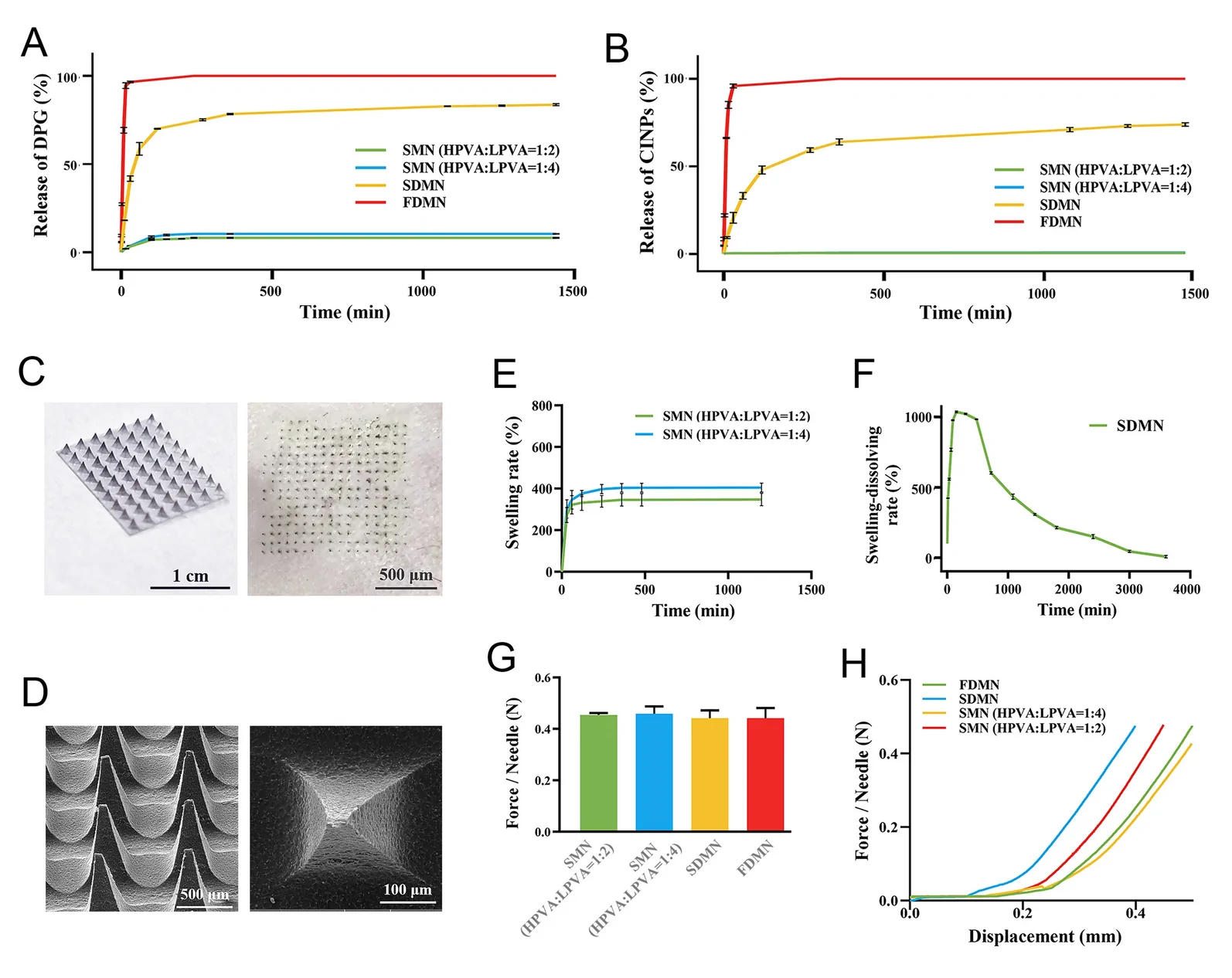
Characterization of MNs. Release profile of DPG (**A**) and CINPs (**B**) from various MNs formulations. **C** Representative macroscopic image of MNs array and the melanin left in mouse skin after MNs insertion. **D** Representative TEM images of MNs array and single needle. **E** Swelling profile of SMN. **F** Swelling–dissolving profile of SDMN. **G** Maximum breaking strength of different formulations of MNs. **H** Force–displacement profiles of different formulations of MNs. Data are expressed as the mean ± SD (*n* = 3), *P* values are calculated using one-way ANOVA with Bonferroni correction, n.s.: *P* > 0.05, *P < 0.05, **P < 0.01, ***P < 0.001

To develop microneedle tips with distinct drug release profiles, SMN, SDMN and FDMN were prepared based on previous work (Shi et al. 2022). HPVA and LPVA exhibit differentiated degrees of alcoholysis, with lower alcoholysis corresponding to enhanced swelling properties of PVA. Therefore, MNs with different swelling properties could be obtained by changing the ratio of HPVA and LPVA. SMN was prepared using PVA with different ratio of HPVA and LPVA under a total concentration of 20%. LPVA and PVP were used to fabricate SDMN with a total concentration of 20%. FDMN was fabricated by 35% PVP. The prepared MNs were uniform and structurally intact, arranged in 40 × 40 arrays on 20 × 20 mm substrate. Each MNs exhibited a pyramidal shape with a base edge length of 300 μm and a height of 600 μm (Fig. 5D). The compressive strength exceeded 0.4 N per needle, indicating that the MNs were mechanically robust enough to penetrate the skin (Fig. 5G, H), as confirmed by the appearance of black dots on the skin following insertion (Fig. 5C).

The drug release profiles and dissolving behaviors of different MNs were then evaluated. SMN reached swelling equilibrium within 10 h and maintained its swollen state for at least 24 h. The swelling rate exceeded 300% (Fig. 5E), indicating that SMN possessed excellent swelling properties that conferred with enhanced intradermal retention. SDMN reached swelling equilibrium quickly within 150 min and then dissolved slowly within 2.5 days (Fig. 5F), which enabled SDMN a sustained drug release profile. It showed that the needles of SMN could maintain their morphology within seven day observation, similar with previous study (Supplementary Fig. S2A). However, SMN released less than 5% of DPG (Fig. 5A) and no more than 1% CINPs within 24 h (Fig. 5B), which might be attributed to the dense mesh structure of PVA. In contrast, SDMN released 80% of DPG (Fig. 5A) and approximately 70% of CINPs (Fig. 5B) in 24 h, whereas FDMN completely released both DPG and CINPs within a few minutes (Fig. 5A, B). Given the inflammatory microenvironment and oxidative stress present in vitiligo lesions, a faster drug release profile is desirable. Therefore, SDMN and FDMN were selected for subsequent experiments.

### Vitiligo mouse model and in vivo therapeutic efficacy assessment

**Fig. 6:**
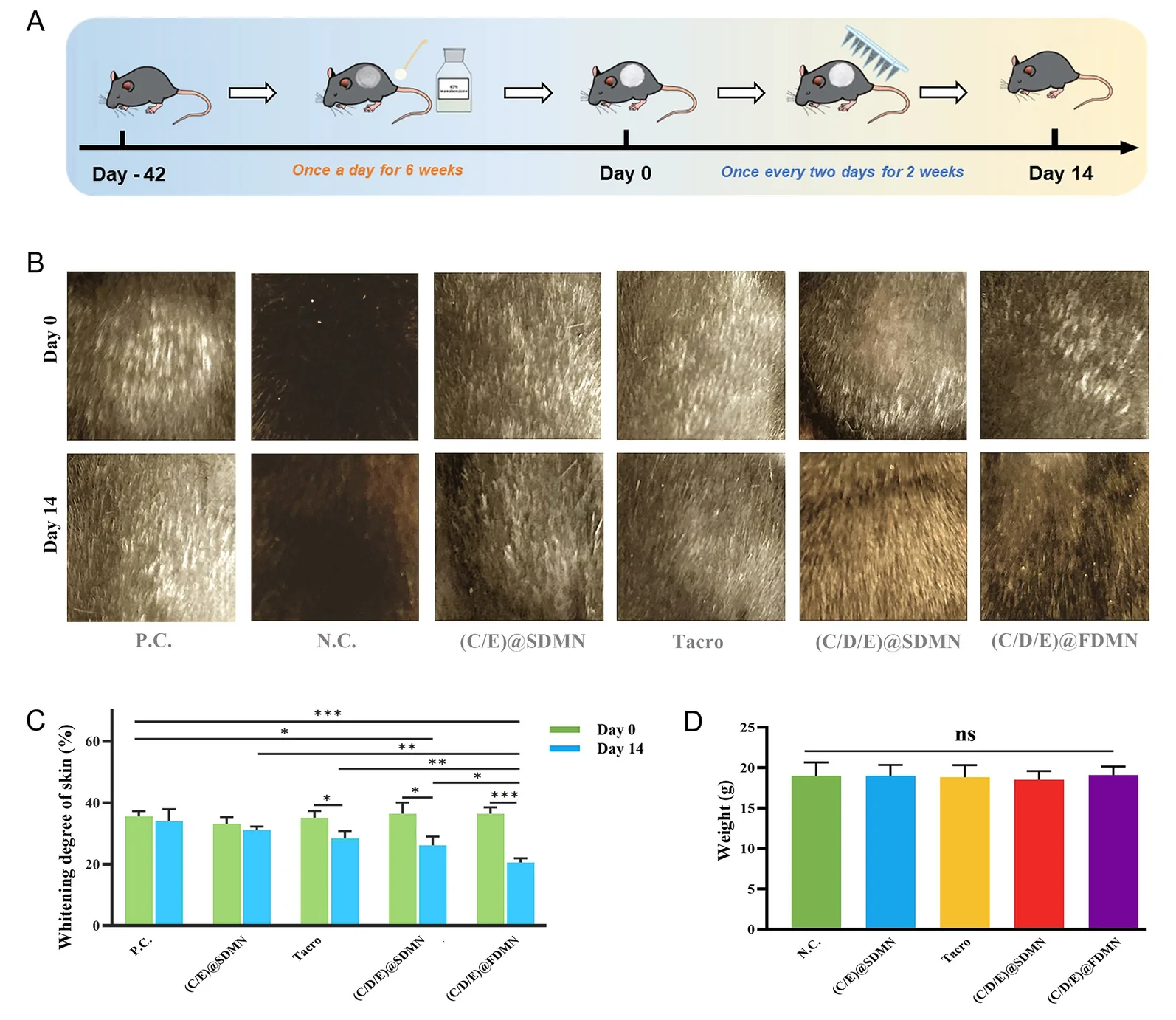
In vivo therapeutic efficacies assessment of microneedle on vitiligo mice model. **A** Schematic diagram of mouse vitiligo model establishment and therapy protocol. **B** Comparison of hair color of mice before and after treatment of tacrolimus ointment, (C/E)@SDMN, (C/D/E)@SDMN or (C/D/E)@FDMN. The untreated vitiligo mice were taken as positive control (P.C.) and the normal mice were taken as negative control (N.C.). **C** Quantitative analysis of skin whitening degree of mice before and after treatment. **D** Body weight of mice at the end of various treatments. Data are expressed as the mean ± SD (*n* = 3), *P* values are calculated using one-way ANOVA with Bonferroni correction, n.s.: *P* > 0.05, *P < 0.05, **P < 0.01, ***P < 0.001

**Fig. 7:**
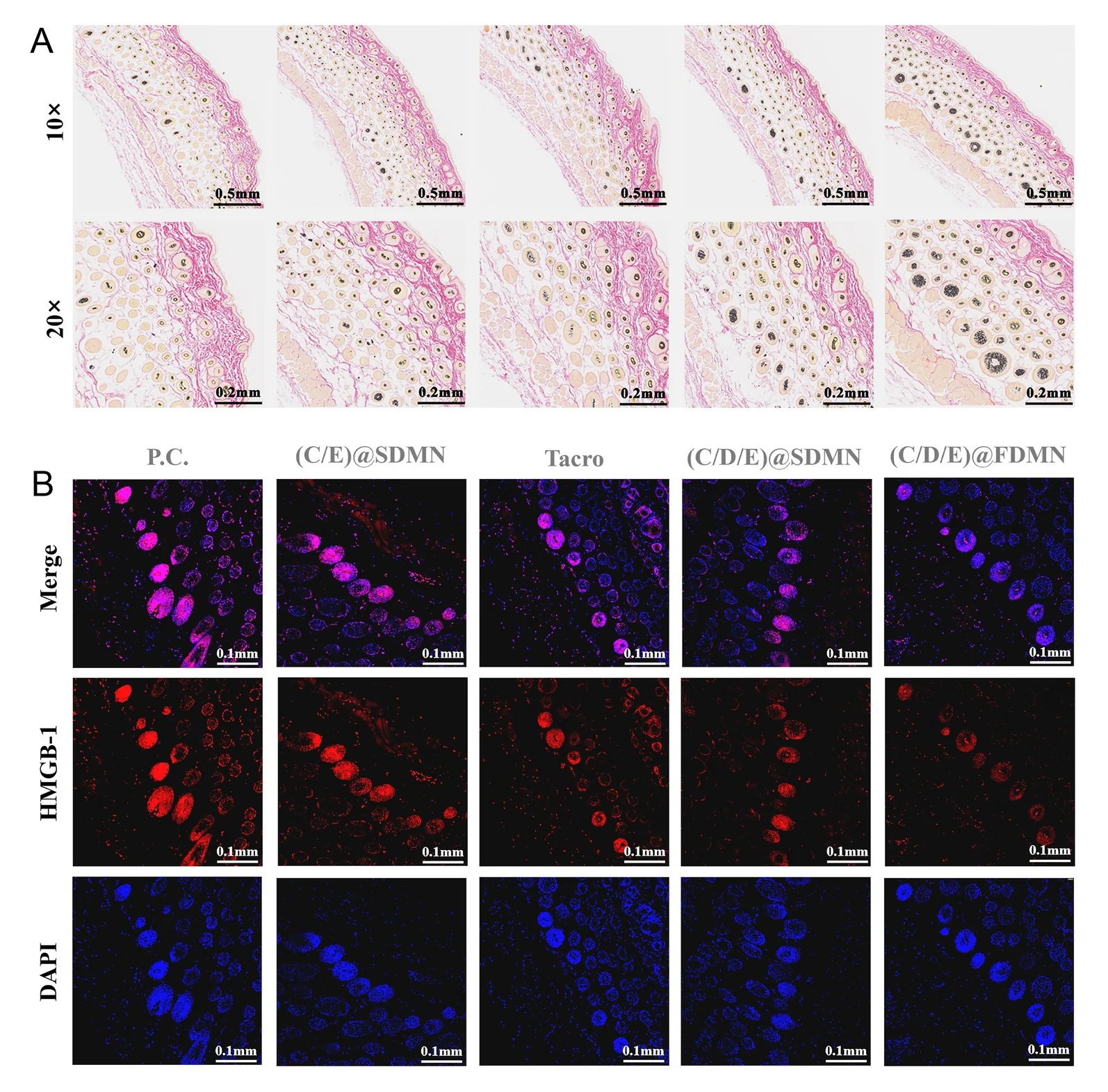
Representative images of tissue section staining after treatments on vitiligo mice model. **A** Masson–Fontana staining of mice skin section after various treatments. **B** Immunofluorescence staining of mouse skin section, red indicated HMGB-1, blue indicated nucleus, purple indicated merged areas

A vitiligo mouse model was successfully established by applying monobenzone ointment onto the backs of *C57BL/6* mice for six weeks. To evaluate the in vivo efficacy of MNs, shaved mice were treated with tacrolimus ointment or MNs insertion. As shown in Fig. 6A, after the mice were shaved, (C/E)@SDMN, (C/D/E)@SDMN, (C/D/E)@FDMN were inserted into the vitiligo area of the mice at a frequency of once every two days, the tacrolimus ointment was applied at the same frequency. The results showed that (C/E)@SDMN was less effective than the first-line drug tacrolimus ointment, whereas both (C/D/E)@SDMN and (C/D/E)@FDMN demonstrated superior efficacy compared to tacrolimus. Hair repigmentation assessments revealed that tacrolimus, (C/D/E)@SDMN and (C/D/E)@FDMN groups all significantly reduced hair whitening areas (Fig. 6B), which was also reflected by the quantitative analysis (Fig. 6C). Masson–Fontana melanin staining revealed that the untreated mice exhibited minimal melanin content in the skin, while the treated groups showed increased melanin levels to varying degrees, there into the (C/D/E)@FDMN group demonstrated the most pronounced effect (Fig. 7A). Immunofluorescence staining of HMGB-1 in tissue sections showed higher levels of HMGB-1 in the untreated mice skin than those in the treated groups (Fig. 7B). Notably, the (C/E)@SDMN group showed less effect on repigmentation, melanogenesis and HMGB-1 inhibition than the (C/D/E)@SDMN group, suggesting the anti-inflammatory effect of DPG was necessary for vitiligo treatment. Furthermore, no significant differences in body weight were observed among the groups by the end of treatment (Fig. 6D), further confirming the safety of the microneedle therapy. These results demonstrated that the precise and efficient delivery of active ingredients to vitiligo lesions via (C/D/E)@FDMN significantly inhibited inflammatory cytokine secretion, effectively activated melanogenesis, and achieved efficient repigmentation, altogether synergistically, safely and markedly alleviated vitiligo symptoms and improved treatment outcomes.

## Discussion

### Transdermal drug delivery for vitiligo treatment

Vitiligo is a chronic skin disorder characterized by the destruction of melanocytes and loss of local pigment. Its pathogenesis remains complex that might be correlated with autoimmune responses, oxidative stress and genetic predisposition (Chen et al. 2021). For vitiligo, transdermal drug administration is particularly advantageous by direct efficacies and improved patient compliance (Garg et al. 2016). Nevertheless, transdermal drug delivering efficiency is greatly impeded by the stratum corneum. Microneedles could create microchannels across the stratum corneum and dramatically enhance transdermal drug penetration and bioavailability, which has been validated using various vitiligo drugs with more favorable therapeutic efficacies (Ebrahim and Albalate 2020; Nofal et al. 2022). With the aid of microneedles, large bioactive molecules, such as alpha-melanocyte-stimulating hormone (α-MSH), tofacitinib or polydopamine melanin could also be transdermally delivered to restore pigmentation in vitiligo mouse models (Li et al. 2025; Liang et al. 2023). Notably, the drug release profiles would determine the therapeutic outcomes. In this study, microneedles with different dissolving/swelling properties were fabricated, namely, fast-dissolving MNs (FDMN), swellable MNs (SMN) and slow-dissolving MNs (SDMN). FDMN was able to achieve immediate in vitro drug release (Fig. 4A, B) and displayed significantly lower whitening degree than SDMN in vivo (Fig. 6C). The superiority of FDMN over the other formulations highlighted the necessity of rapid drug release to effectively counteract the dynamic inflammatory and oxidative cascades in vitiligo therapy, especially for CINPs. For one thing, the particle size of CINPs was relatively larger than the micropore size within swelling SMN needles. For another, the abundant functional groups on the CINPs surface may interact with PVA. These factors collectively contribute to the slow-release profile of CINPs from SMN and SDMN. Such comparison study provides valuable instructions on selecting microneedle formulations based on specific drugs for vitiligo treatment.

### Synergetic regulation of vitiligo microenvironment

Oxidative stress plays crucial roles in vitiligo onset, while autoimmunity mainly contributes to disease progression (Toosi et al. 2012). Oxidative stress triggers the release of HMGB-1 from MCs, which facilitated the secretion of CXCL-16 and IL-8 from keratinocytes, leading to the formation of chemotaxis for recruiting autoreactive cytotoxic CD8^+^ T cells (Cui et al. 2019). CD8^+^ T cells’ specific attack resulting into massive MCs death, skin pigmentation loss and the formation of white patches (Passeron et al. 2022). Therefore, alleviation of the oxidative stress and reducing recruitment of CD8^+^ T cells are essential to reverse the adverse vitiligo microenvironment.

In this study, CINPs, DPG and EXO were applied to improve the vitiligo microenvironment. Thereinto, CINPs offered dual benefits of antioxidant activities and melanin supplementation. The structural homology of CINPs with mammalian melanin ensured them to be integrated into the epidermal pigment system, which was evidenced by in vitro efficient internalization of HaCaTs (Fig. 3) and in vivo rapid repigmentation (Fig. 7A). DPG served as an inhibitor of HMGB-1, together with CINPs to exert anti-inflammatory and anti-chemotaxic effects. In vitro cytokine assessment results were consistent with the postulation that CINPs + DPG group generated the lowest level of IL-8, HMGB-1 and CXCL-16 (Fig. 4B). Furthermore, the incorporation of mouse skin derived EXO significantly enhanced both HaCaTs and MCs migration and proliferation, underscoring its key effects on restoring melanocyte populations in vitiligo lesion (Supplementary Fig. S6). Through the synergetic effects of alleviating oxidative stress, suppressing inflammation and promoting melanocyte proliferation and repigmentation, the adverse microenvironment was significantly improved. The whitening degree of vitiligo mice model reduced by 15.5% after treatment with C/D/E@MNs, significantly higher than 6.8% of tacrolimus. C/D/E@MNs therefore provided a safe and promising alternative for vitiligo therapy.

### Further considerations for improvements

The FTIR spectra of CINPs and standard melanin are not entirely consistent, except for the characteristic absorption peaks at 3405 cm⁻^1^ and 1621 cm⁻^1^. Cuttlefish ink components primarily consist of water, melanin, crude proteins, carbohydrates, and crude lipids. The purification method used in the experiment can only remove water-soluble impurities from ink. Enzymatic digestion effectively eliminates impurities such as melanin-binding proteins without compromising the structural integrity of melanin. In contrast, hydrochloric acid hydrolysis removes proteins, polysaccharides, lipids, and other components but induces chemical modifications with functional groups on the melanin surface, thereby compromising its surface morphology (Xie et al. 2021). Future studies should focus on refining the extraction protocols for CINPs to enhance sample purity while maintaining their bioactive properties.

Emerging researches highlight that exosomes have therapeutic potential in modulating the pathological microenvironment of vitiligo in addition to their fundamental biological functions on regulating cellular proliferation, migration, and tissue regeneration. In vitiligo lesions, overexpression of PTEN induces oxidative stress in melanocytes, subsequently triggering apoptosis. Zhu and colleagues revealed that co-culturing human melanocytes with mesenchymal stem cells in a transwell system promoted melanocyte proliferation and suppressed PTEN expression, thereby mitigating oxidative stress-induced apoptosis (Zhu et al. 2020). Wang and colleagues demonstrated that 3D-engineered exosomes derived from human umbilical cord–mesenchymal stem cells (hUC–MSCs) ameliorate depigmentation in vitiligo mouse model by enhancing Treg cell-mediated immunosuppression, suppressing oxidative stress-induced melanocyte damage, reducing CD8⁺T cell infiltration, and elevating cutaneous Treg cell populations (Wang et al. 2024). Building on these findings, future research could focus on the precise mechanisms through which exosomes exert their therapeutic effects in vitiligo, particularly their molecular pathways for melanocyte protection and immune regulation, to accelerate clinical translation of exosome-based therapies.

Identifying a mouse model that more accurately replicates the pathogenic features of human vitiligo has long been an issue to be addressed. Beyond the monobenzone-induced model, protocols including topical application of hydrogen peroxide (Sun et al. 2021) or endogenous autoreactive CD8⁺ T cells targeting skin melanocytes (Chen et al. 2022) have been developed. However, there is currently no definitive consensus regarding which model most genuinely mimics vitiligo. Skin organoids, in vitro 3D tissue constructs comprising various cell types and exhibiting morphological and functional competence, has been developed and applied as preclinical patient-derived tumor xenograft models, atopic dermatitis models or psoriasis models (Hong et al. 2023). They are increasingly recognized as effective alternatives to traditional culture and human skin due to their abilities to overcome the limitations of two-dimensional systems and ethical concerns (Zhang et al. 2024). Vitiligo organoids accordingly provide promising potential for developing eligible model to study pathological mechanisms and therapeutic interventions.

CINPs as natural melanin were first applied to validate their therapeutic effects on vitiligo. Although C/D/E@FDMNs exerted obviously therapeutic outcomes via their fast-dissolving property, it was unsuitable to maintain a long-acting effects, especially for vitiligo such a chronic disease requiring long-term treatment. Therefore, high administration frequency was necessary during the treatment, which may potentially cause secondary damage to the lesion sites. Hydrogel-based swellable MNs displayed sustained drug release capabilities (Yu et al. 2021), but their pore size and swelling property require to be optimized, allowing for sustained and effective release of CINPs. Moreover, the underlying molecular mechanisms of C/D/E@FDMNs in improving vitiligo treatment also need further investigations.

## Conclusion

In this study, a comprehensive (C/D/E)@MNs with exogenous melanin supplementation, improvement of the inflammatory microenvironment, and promotion of melanocyte migration and proliferation was fabricated for vitiligo treatment. By adjusting the needle solution, MNs can quickly dissolve and efficiently deliver the active ingredients after insertion into the skin, realizing precise, rapid and efficient delivery to the vitiligo lesion. Compared with topical administration of tacrolimus ointment, (C/D/E)@FDMN significantly alleviated vitiligo symptoms and promoted the repigmentation of the lesion sites. This efficient, precise, and self-administered platform represents a promising therapeutic alternative for vitiligo patients.

## Supplementary Information

Below is the link to the electronic supplementary material.Supplementary file1 (DOCX 17525 KB)

## Data Availability

The data used to support the findings of this study are available from the corresponding author upon request.
