# Supplementary material for: Design and construction of cuttlefish ink derived melanin complexed microneedles for microenvironment regulation to improve vitiligo treatment
Source: Mar Life Sci Technol. 2025 Aug 6;8(3):798–809. doi: 10.1007/s42995-025-00318-5 (PMC13525130; doi:10.1007/s42995-025-00318-5)
Supplement: Supplementary file 1 [file 42995_2025_318_MOESM1_ESM.docx]

**Supplementary Information**

**Design and construction of cuttlefish ink derived melanin complexed microneedles for microenvironment regulation to improve vitiligo treatment**

Weimiao Li ^a1^, Yan Shi ^a1^, Ruimin Zhan^a^, Lu Liu^a^, Jiarui Wang^a^, Minhyeock Lee^c^, Bingqiang Zhang^d^, Shaoshuai Liang^d^, Zhiguo Wang^b^, Ming Kong^a*^

^a^ College of Marine Life Science, Ocean University of China, Qingdao, 266003, China

^b^ Department of Burn and Plastic Surgery, the Affiliated Hospital of Qingdao University, Qingdao University, Qingdao, 266003, China

^c^ Department of Biotechnology, College of Life Sciences and Biotechnology, Korea University, Seoul, 02841, Republic of Korea

^d^ Qingdao Restore Biotechnology Co., Ltd., Qingdao, Shandong 266111, P.R. China

^*^ Corresponding author.

E-mail address: [kongming@ouc.edu.cn](mailto:kongming@ouc.edu.cn)

^1^ These authors contributed equally to this article.

**Materials and methods**

**Extraction and primary culture of** **dorsal skin cells**

*C57BL/6* mice were anesthetized and sacrificed using cervical dislocation. The hair on dorsal skin was completely removed. The mice were immersed in 75% ethanol for 15 min, and the dorsal skin was dissected carefully under sterile conditions. After removing the blood vessels and fat tissue, the skin was rinsed using PBS supplemented with penicillin and streptomycin. The skin tissue was finely scissored and distributed at the base of a cell culture flask filled with DMEM containing 20% FBS, then cultured under 37°C and 5% CO_2_ for 72 h. After migrating from the tissue, the cells were expanded and cultured in DMEM containing 10% FBS and penicillin/streptomycin (100 U mL^-1^, 100 U mL^-1^).

**Extraction and characterization of exosomes**

Exosomes of dorsal skin cells were isolated from low-passage-number skin cells of primary culture through ultracentrifugation. The supernatants of cell culture were collected and subjected to sequential centrifugation, 300 🞨g for 10 min, 2000 🞨g for 10 min and 10000 🞨g for 30 min to remove the deposit. Finally, the supernatant was centrifuged at 100000 🞨g for 70 min. The pellet was resuspended in PBS and centrifugated again to purify the exosomes. All centrifugation steps were performed at 4°C. The exosome suspension in PBS was stored at -80°C for further use. The concentration of exosomes was determined using a BCA assay kit, the size and zeta potential were measured using Zetasizer, and the morphology were observed by TEM.

**Extraction and primary culture of melanocytes (MCs)**

MCs were extracted from *C57BL/6* mice dorsal skin. The depilated dorsal skin was dissected, which was rinsed thoroughly using HBSS containing penicillin and streptomycin, and then placed into conical tubes containing 0.25% Dispase II. The skin was incubated at 4°C overnight. The epidermal tissue was peeled off carefully, trimmed into fine pieces and digested with 0.25% trypsin-EDTA for 5 min at room temperature. DMEM containing 10% FBS was added and gently mix with sample. The dissociated cells were collected by centrifugation at 150 🞨g for 5 min, resuspend in DMEM containing 10% FBS and incubate under 37°C, 5% CO_2_.

***In vitro* cell viability assay**

L-929 cells were inoculated in 96-well plates at a density of 1×10^5^ cells mL^-1^. After 24 h incubation, the medium was discarded and replaced with new medium supplemented with different materials (HPVA, LPVA, PVP, CINPs, DPG) at various concentrations. The untreated group was taken as the negative control (N.C.). Cell proliferation and cell viability were detected using Cell Counting Kit-8 (CCK-8). The survival of the cells was detected by Live/Dead viability assay kit.

**Establishment of oxidative stress cell model**

HaCaTs were inoculated in 96-well plates at a density of 1×10^5^ cells mL^-1^. The control group was HaCaTs without H_2_O_2_ treatment. H_2_O_2_ was added in each well of the experimental group at concentrations of 0.25 mM, 0.5 mM, 0.75 mM, 1.0 mM, 1.5 mM or 2.0 mM, respectively. The cells were incubated under 37°C and 5% CO_2_ for 24 h. The cell viability was determined by CCK-8 kit, the concentration of H_2_O_2_ at which 50% cells were viable was taken for the establishment of the oxidative stress model of HaCaTs.

**Fig. S1**. Representative microscopic images of primary culture of mouse dorsal skin cells (A) and mouse melanocytes (B). Size distribution (C) and zeta potential (D) of EXO derived from primary culture of mouse dorsal skin cells.

**Fig. S2**. (A) Representative images of dissolving and degrading performances of SMN in simulated body fluid at different time intervals. (B) Insertion assessment of CINPs loaded SDMN into a simulated skin (agarose hydrogel, 3% m/v) and the magnified field of the inserted area. (C) Insertion ratio assessment of SDMN into mouse dorsal skin stained with trypan blue to show the pinholes. (D) Insertion assessment of CINPs loaded FDMN into a simulated skin and the magnified field of the inserted area. (E) Insertion ratio assessment of FDMN into mouse dorsal skin stained with trypan blue.

**Fig. S3**. Representative live-dead staining images of L929 cells incubated with different concentrations of HPVA, LPVA, PVP, DPG or CINPs for 24 h (scale bar = 200 μm).

**Fig. S4**. (A) Relative growth rate of L929 cells incubated with different concentrations of HPVA, LPVA, PVP, CINPs or DPG for 24, 48 or 72 h, respectively. (B) Hemocompatibility assessment of different concentrations of HPVA, LPVA, PVP, CINPs or DPG.

**Fig. S5**. Cell viability of HaCaTs incubated with different concentrations of H_2_O_2_ for 24 h.

**Fig. S6**. (A) Conﬂuent monolayers of MCs were scratched with a pipette tip and incubated with CINP (60 μg mL^-1^), CINP +DPG (60 μg mL^-1^ CINP and 300 μg mL^-1^ DPG) or CINP +DPG+EXO (60 μg mL^-1^ CINP, 300 μg mL^-1^ DPG and 60 μg mL^-1^ EXO), respectively. The untreated MCs was taken as N.C.. (B) Quantitative analysis of the decrease in scratching area of MCs. (C) Relative growth rate of MCs incubated with CINP (60 μg mL^-1^), CINP +DPG (60 μg mL^-1^ CINP and 300 μg mL^-1^ DPG) or CINP +DPG+EXO (60 μg mL^-1^ CINP, 300 μg mL^-1^ DPG and 60 μg mL^-1^ EXO) , respectively for 24 h. The untreated MCs was taken as N.C.. Data are expressed as the mean ± SD (n = 3), p-values are calculated using one-way ANOVA with Bonferroni correction, n.s.: P > 0.05, ∗P < 0.05, ∗∗P < 0.01, ∗∗∗P < 0.001.
